# Rapid detection of wheat yellow mosaic virus by reverse transcription loop-mediated isothermal amplification

**DOI:** 10.1186/1743-422X-8-550

**Published:** 2011-12-20

**Authors:** Zong-Ying Zhang, Xiao-Jun Liu, Da-Wei Li, Jia-Lin Yu, Cheng-Gui Han

**Affiliations:** 1 State Key Laboratory for Agrobiotechnology and Department of Plant Pathology, China Agricultural University, Beijing 100193, China

**Keywords:** Wheat yellow mosaic virus, RT-LAMP, Virus detection

## Abstract

For the detection of wheat yellow mosaic virus (WYMV), we established a reverse transcription loop-mediated isothermal amplification (RT-LAMP) method. Using Primer Explorer software, four sets of primers were designed and RT-LAMP assay reaction conditions were optimized. The RT-LAMP was performed at different times by four primer sets. Agarose gel analysis showed that WYMV could be detected after 30 min with the primer set III and after 45 min with the other three primer sets, both under the 80-min reaction time. RT-LAMP had the same results with the four primer sets, thus primer set III and 65°C for 80 min reaction were selected for virus detection. There was no significant different when avian myeloblastosis virus (AMV) and moloney murine leukemia virus (M-MLV) RT-LAMP with the four primer sets and M-MLV was chosen due to its relatively cheap price. The result on specificity showed that the assay could amplify WYMV specifically, and the sensitivity comparison showed that the RT-LAMP was 100 times more sensitive than conventional reverse-transcriptase-polymerase chain reaction (RT-PCR). Overall, RT-LAMP was found to be a simple, specific, sensitive, convenient and time-saving method for WYMV detection.

## Background

Wheat yellow mosaic is one of the most devastating soil-borne diseases of winter wheat (*Triticum aestivum *L.). It was first reported in Japan in the 1920s and China in the 1960s [1,2], and then spread continually in Japan and China [3,4]. According to the statistical data, the disease area was more than 666,700 hectares in the 1990s, the yield loss was estimated to range between 20-40% and could be up to 70-80% during a serious year, even 100%.

*Wheat yellow mosaic virus *(WYMV), the causal agent of wheat yellow mosaic, belongs to the genus *Bymovirus *within the family *Potyviridae*. It is a soil-borne pathogen and is transmitted by the fungus-like organism *Polymyxa graminis *[5]. The genome of WYMV is comprised of two (+) single-stranded RNAs, RNA1 encodes for coat protein (CP) and six others: P3, 7 K, nuclear inclusion protein a (NIa), nuclear inclusion protein b (NIb), cytoplasmic inclusion protein (CI), 14 K; RNA2 encodes for a polyprotein that contains 28-kDa and 72-kDa proteins [6,7].

Concerning virus detection, several methods are used commonly to detect WYMV. ELISA is a reliable method for detecting WYMV and suitable for high-throughput samples [8-10]; RT-PCR is the most conventional method to detect RNA virus [11,12] and western blotting detects the target protein for further confirmation [13-16]. However, the sensitivity of ELISA might not be sufficiently high to detect low concentrations of WYMV, and virus-specific antiserum is required. WYMV can serologically cross-react with wheat spindle streak mosaic virus [17], and RT-PCR is not perfect either.

Novel nucleic acid amplification methods, loop-mediated isothermal amplification (LAMP) for DNA and RT-LAMP for RNA, have been developed [18]. The high specificity and sensitivity, rapid execution, performance under isothermal condition, time-saving, easy observation of by-products [19], and low cost make RT-LAMP unrivaled among diagnostic techniques. It is easy and simple to perform only with four appropriate primers, a reverse transcriptase for RNA template, a DNA polymerase and a water bath or heat block for reaction. Therefore, in recent years, many pathogenic viruses have been detected by these methods, including human [20-24], animal [25] and plant [26-32] viruses.

In the present study, the RT-LAMP method was used successfully for detection of WYMV for the first time. This method could result in more accurate diagnosis for monitoring WYMV.

## Materials and methods

### Plant materials

Wheat samples were collected during field surveys from different regions of China in March 2011 and stored in a freezer at-20°C. The Chinese wheat mosaic virus (CWMV)-infected samples were collected from Yantai, Shandong Province; the barley stripe mosaic virus (BSMV)-infected samples were fresh wheat leaves inoculated with BSMV in our laboratory in May 2011.

### Total RNA extraction

The fresh or stored wheat samples were ground in a Retsch MM400 mixer mill (Retsch, Haan, Germany) for 1 min at 30 Hz, the sample powder was homogenized with 600 μl extraction buffer (0.1 M Tris-HCl, pH 7.4, 2.5 mM NaCl, and EDTA) and 600 μl supercritical water-phenol, then centrifuged at 12,000 rpm for 15 min. The aqueous phase was precipitated with 4 M LiCl. After incubation at -20°C for 2 h or overnight, the precipitate was collected by centrifugation (12,000 rpm) at 4°C for 15 min. The resultant pellet was washed twice with 70% ethanol, dried at 37°C for about 5 min, and dissolved in 60 μl deionized distilled water. The RNA extract was stored at -20°C.

### Primer design

Based on published WYMV RNA1 and RNA2 sequences (accession numbers AF067124 for RNA1and AF041041 for RNA2) [7], four sets of primers were designed by Primer Explorer version 4 (Fujitsu Ltd., Tokyo Japan, http://primerexplorer.jp/elamp3.0.0/index.html). Four oligonucleotide primers [F3, B3, FIP (F1c + F2), and BIP (B1c + B2)] that recognize a total of six sequences of the CP gene and the 72 kDa gene were designed, respectively. F3 and B3 were outer primers whereas FIP and BIP are inner primers. Each inner primers has two distinct adjacent sequences in opposite orientations. All primers were PAGE purified and synthesized by Invitrogen or Sanggon (Shanghai, China).

### RT-LAMP detection

To choose the most appropriate primer set, RT-LAMP reactions were conducted as described previously [18,19,33]. The reaction was carried out in a 25-μl reaction system, containing 2.5 μl 10× Thermopol buffer, 0.5 mM dNTP, 0.8 M betaine, 1.6 μM FIP and 1.6 μM BIP, 0.2 μM F3 and 0.2 μM B3, 0.45 mM MgCl_2 _and 1.8 mM DTT, 4U RNase Inhibitor (TaKaRa, Biotechnology, Dalian, China), 200 U moloney murine leukemia virus (M-MLV) transcriptase or 1.25 U avian myeloblastosis virus (AMV) reverse transcriptase (Promega, Madison, WI, USA), 8 U *Bacillus stearothermophilus *(*Bst*) DNA polymerase (New England Biolabs, Ipswich, MA, USA), 1.0 μl RNA extract and 4.0 μl deionized distilled water. Four sets of primers (I-IV) located in the CP and the 72 kDa gene, were used to detect WYMV at 65°C for 25-80 min, and to compare RT-LAMP in the presence of M-MLV or AMV reverse transcriptase. The final products of RT-LAMP were a mixture of stem-loop DNAs with various stem lengths and cauliflower-like structures with multiple loops. For WYMV-positive sample, the linearized DNA form showed up in the lane by agarose gel analysis. Many pyrophosphate ions were produced during RT-LAMP, and the production of a white precipitate of magnesium pyrophosphate gave the tube a turbid appearance that could be observed directly. The positive tube was cloudy and the negative one was clear. With four sets of primers, the RT-LAMP reaction mixture was observed by the naked eye, and the amplification reactions were confirmed by complementary procedures, such as gel electrophoresis. Five microliters of amplified DNA fragments were electrophoresed in 1.5% agarose in TBE buffer.

### Conventional RT-PCR detection

The RT mixture contained 2 μl RNA extract, 0.5 μl primer (Table 1), 3 μl 5 × M-MLV buffer and 1 μl dNTP (5 mM each), 12 U RRI, 200 U M-MLV reverse transcriptase (Promega, Madison, WI, USA) and 7.5 μl deionized distilled water, added to a final volume of 15 μl. Reverse transcription was performed at 37°C for 1.5 h, and 2.0 μl product of cDNA was used for PCR amplification. Twenty-five microliters of PCR mixture that contained 2.5 μl PCR buffer, 0.5 μl dNTP (5 mM each), 0.5 μM primers, 1.25 U *Taq *DNA polymerase (Tiangen, Beijing, China), and 21.5 μl deionized distilled water were added. The PCR was performed with denaturation at 94°C for 5 min and 30 cycles of 94°C for 40 s, 63°C for 45 s, and 72°C for 40 s, followed by a final 10-min extension step in a Bio-Rad Cycler(Bio-Rad, Hercules, CA, USA). Five microliters of amplified DNA fragments were electrophoresed in 1.5% agarose in TBE buffer.

**Table 1:** Primers used for RT-LAMP and RT-PCR

	Primer	Type	Target gene	Sequence(5'-3')	**Genome position**^**a**^
	F3-1	Forward outer		TGAAACACGGCGCATCTG	665-682
			
I	B3-1	Reverse outer	*CP*	AGTTCTGGGTGTCCATCAGT	875-856
			
	FIP-1	Forward inner		GCAACTTCGATGTCCTGTGGGTCCGCGTACGCTTTTGACT	752-731,689-706
			
	BIP-1	Reverse inner		AGCACGTCTTGCTGCTTTAGGCGGTTGTCTTGCGGAGGTT	759-780,837-820

	F3-2	Forward outer		GCAGATCGTGTTGAGGCC	106-123
			
II	B3-2	Reverse outer	*CP*	ATAGATTTGGGTGCGCTCTT	332-313
			
	FIP-2	Forward inner		CCAGCGTCGGCAACAATTTTGCAGCGATGACAAGAAAGCCAG	212-191,142-161
			
	BIP-2	Reverse inner		CAAAAAGGACCAATGCCGCCACGAAGCTTTAGTCCTGCGTT	215-235,293-274

	F3-3	Forward outer		ACTACCATTCTCGCAGCACT	715-734
			
III	B3-3	Reverse outer	*72 kDa*	GGTCACGAACGACAGTGC	916-899
			
	FIP-3	Forward inner		GCCGAACCATGGAAAGGTGTCT-TGGCGTTGTTTCCTTAGTGC	813-729,748-767
			
	BIP-3	Reverse inner		TACTCCCACGGAGGTCCTCC-GCTGGTAAGAAGCATCCCG	814-833,889-871

	F3-4	Forward outer		GGGGTTTCGAACACGAATGG	1335-1354
			
IV	B3-4	Reverse outer	*72 kDa*	AGCGAGGCCTCAGCATTA	1517-1500
			
	FIP-4	Forward inner		CATGTCCAGTGCTCTTGCGTCG-CATCCACGCTGCAACGAA	1428-1407,1355-1372
			
	BIP-V	Reverse inner		ACGAGCGAGCTTTTCCAATCCA-CAAGCAGCGAGACAATGTCA	1429-1450,1498-1479

	HC72-157 F	Forward Primer	*72 kDa*	TGCGAAGCCTCTACGACCTGTTT	157-179
		
	HC72-518R	Reverse Primer		TGATGATTGCTCGCCCAACAGA	518-497

	CWMV-F	Forward Primer	*19 kDa*	TATGACTACTGGTACTCA	2652-2669
		
	CWMV-R	Reverse Primer		AATTACTCCACACGAGT	3173-3157

	BSMV-F	Forward Primer	*unknown*	CTTGATGCTTTGGATAAGGCTTA	1864-1887

	BSMV-R	Reverse Primer	protein	AATCTTCCCTTGGGGGAC	2790-2773

^a ^Genome position refers to the nucleotide sequence of WYMV-HC CP and 72 kDa genes (accession number RNA1 AF067124 and RNA2 AF041041), CWMV RNA2 19-kDa gene (accession number AJ012006), and BSMV RNA-gamma segment (accession number M16577)

### Specificity and sensitivity comparison of RT-LAMP and RT-PCR

To determine the specificity of RT-LAMP, total RNA from wheat leaves infected with WYMV, CWMV or BSMV were applied to the RT-LAMP reaction solution separately, and RNA collected from the healthy wheat served as a negative control. The primer sequences used for CWMV and BSMV RT-PCR were shown in Table 1. To compare the sensitivity of RT-LAMP with RT-PCR, total RNA from WYMV-infected wheat was diluted serially in 10-fold increments (10^0^-10^-8^) with healthy wheat RNAs were then used as templates for the two assays. The products were analyzed by agarose gel electrophoresis (1.5% agarose, TBE).

## Results

### Primer design and selection of assay reaction conditions

**Figure 1:**
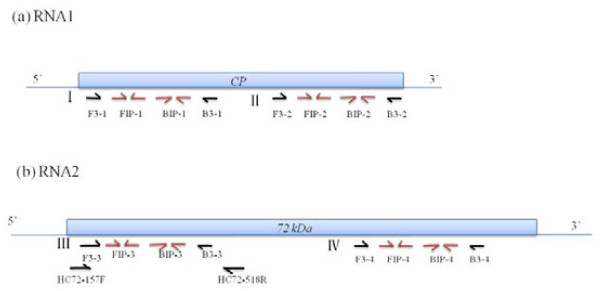
**Primer locations**. Four sets of primers were designed, named I-IV, which targeted the CP (RNA1) gene and 72 kDa (RNA2) gene of WYMV. HC72-157 F and HC72-518R were the conventional RT-PCR primers

Four sets of RT-LAMP primers were designed (I-IV). Primer sets I and II were located at 665-875 nt (3' terminal) and 106-332 nt (close to 5' terminal) of the CP gene; and primer sets III and IV were located at 715-916 nt (close to 5' terminal) and 1335-1557 nt (close to 3' terminal) of the 72 kDa gene (Figure 1).

Each set contains four primers, F3 and B3 were outer primers whereas FIP and BIP were inner primers. Each of the two inner primers had two distinct adjacent sequences in opposite orientations. For detecting CP gene, the first sequence (nt 752-731) of FIP-1 and sequence (nt 212-191) of FIP-2 were in reverse orientation, whereas the second sequence (nt 689-706) and sequence (nt 142-161) of the primers were in the forward direction. In BIP-1 and BIP-2, the forward sequence (nt 759-780) and sequence (nt 215-235) is followed by the reverse sequence (nt 813-729) and sequence (nt 293-274). For the 72 kDa gene: the first sequence (nt 813-729) of FIP-3 and sequence (nt 1428-1407) of FIP-4 were in reverse orientation, whereas the second sequence (nt748-767) and sequence (nt 1355-1372) of the primers were in the forward direction. In BIP-3 and BIP-4, the forward sequence (nt 814-833) and sequence (nt 1429-1450) is followed by the reverse sequence (nt 889-871) and sequence (nt 1498-1479). Their relative location in the virus genomes were shown in Table 1.

**Figure 2:**
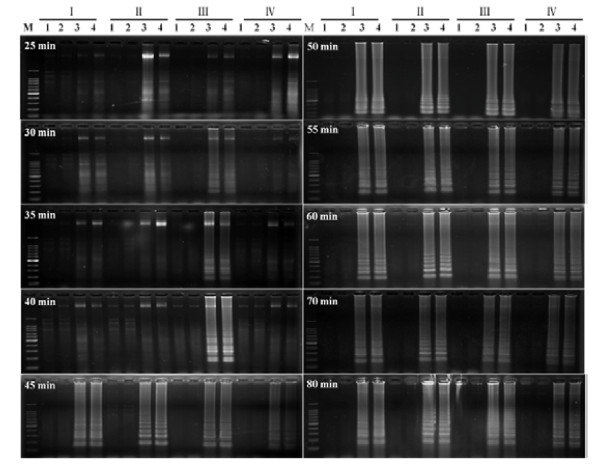
**Agarose gel analysis of RT-LAMP with four primers sets at different reaction times**. Total RNA was extracted from WYMV-infected wheat leaves and detected by RT-LAMP. I-IV were four sets of primers; M: 100-bp DNA ladder marker; 1 and 2: healthy controls; 3 and 4: positive controls

**Figure 3:**
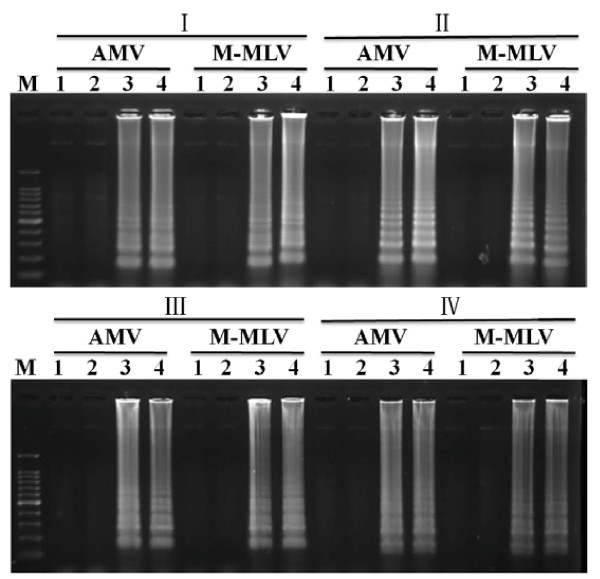
**Agarose gel analysis of different RT-LAMP reactions**. I-IV were four sets of primers; AMV was AMV-mediated RT-LAMP; M-MLV was M-MLV-mediated RT-LAMP. M: 100-bp DNA ladder marker; 1 and 2: healthy controls; 3 and 4: virus-infected samples

To select the appropriate primer set, RT-LAMP using four sets of primers was carried out with two healthy controls and two virus-infected samples under isothermal condition at 65°C for different times. Agarose gel electrophoresis showed that the primer set III had positive bands after 30 min and the other three sets had positive bands after 45 min. They were all available within 80 min, thus the primer set III seemed more sensitive than the others and was selected for virus detection under isothermal conditions at 65°C for 80 min in this study (Figure 2). For each primer set, AMV and M-MLV RT-LAMP was performed and similar results were obtained, therefore, M-MLV reverse transcriptase was chosen for the subsequent RT-LAMP assay because of its relatively cheap price (Figure 3).

**Figure 4:**
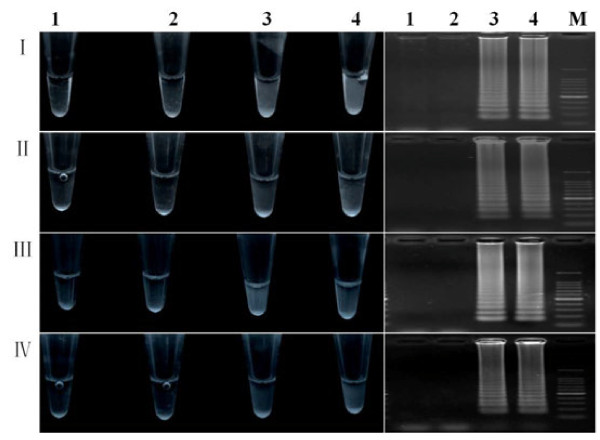
**RT-LAMP reaction mixture turbidity (left) and agarose gel analysis (right)**. I-IV were four sets of primers. M: 100-bp DNA ladder marker; 1 and 2: healthy controls; 3 and 4: WYMV-infected samples

The turbidity of pyrophosphate ions was observed directly after production of large amounts of DNA fragments during the RT-LAMP reaction. RT-LAMP reaction mixtures were used for turbidity observation with the primer sets I-IV after reaction at 65°C for 80 min. The healthy controls were relatively clear, whereas the positive samples were cloudy, and primer sets I and III more readily gave negative or positive results (Figure 4). This demonstrates that the RT-LAMP reactions are conveniently visualized by directly judging the turbidity and it does not require specialized PCR and electrophoresis equipment. The primer set III was chosen for the following experiments.

### Specificity and sensitivity comparison of RT-LAMP and RT-PCR

**Figure 5:**
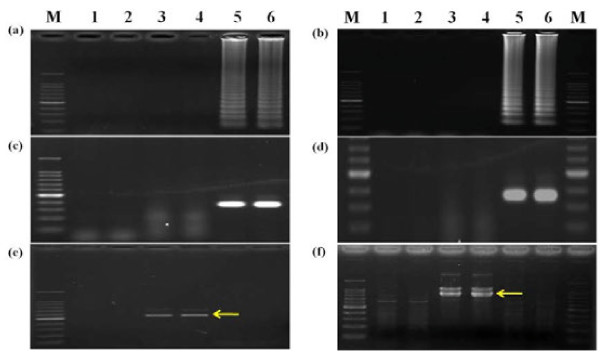
**Specificity of primers for WYMV-infected samples by agarose gel analysis**. (**a**) and (**b**) were RT-LAMP; (**c**) and (**d**) were RT-PCR for WYMV; (**e**) was RT-PCR for CWMV; (**f**) was RT-PCR for BSMV. The arrows showed the CWMV-positive band (left) and BSMV-positive band (right). M: 100-bp DNA ladder marker; 1 and 2: healthy controls; 3 and 4: CWMV-infected wheat leaves (left) and BSMV-infected wheat leaves (right); 5 and 6: WYMV-infected wheat leaves

To evaluate the specificity of RT-LAMP, it was carried out using two healthy controls, two WYMV-infected, two CWMV-infected and two BSMV-infected samples with WYMV RT-LAMP primer set III. At the same time, the samples were also detected by conventional RT-PCR, primers HC-157 F and HC72-518R, which were located at nt 157-179 and 518-497 of the 72-kDa gene, and used to amplify the 360-bp band of interest. The primers CWMV-F and CWMV-R were used for CWMV RT-PCR and primers BSMV-F and BSMV-R were used for BSMV RT-PCR (Table 1). Agarose gel analysis of RT-LAMP revealed that WYMV primers specifically amplified WYMV but not CWMV and BSMV, although CWMV and BSMV could infect wheat with WYMV in some regions of China [12] (Figure 5).

**Figure 6:**
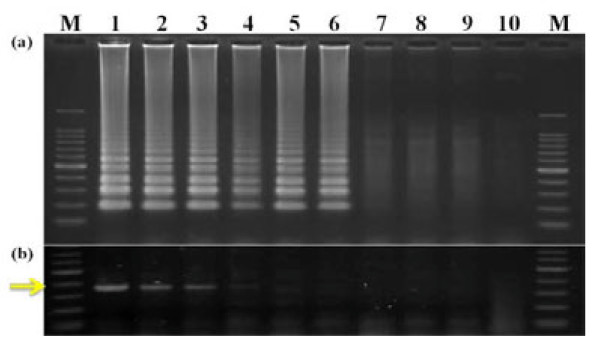
**Comparison of detection sensitivity between RT-LAMP and RT-PCR for WYMV**. Total RNA extract was serially diluted in 10-fold increments (10^0 ^to 10^-8^) with healthy wheat RNAs. The arrow showed the positive band by RT-PCR. M: 100-bp DNA ladder marker; 1-9: total RNA dilution (10^0 ^to 10^-8^); 10: healthy control

The sensitivity comparison showed that RT-LAMP could detect WYMV from total RNA diluted up to 10^-5^, and RT-PCR detected total RNA diluted up to 10^-3^; therefore, RT-LAMP was 100-fold more sensitive than conventional RT-PCR (Figure 6).

### Detection of wheat field samples

**Figure 7:**
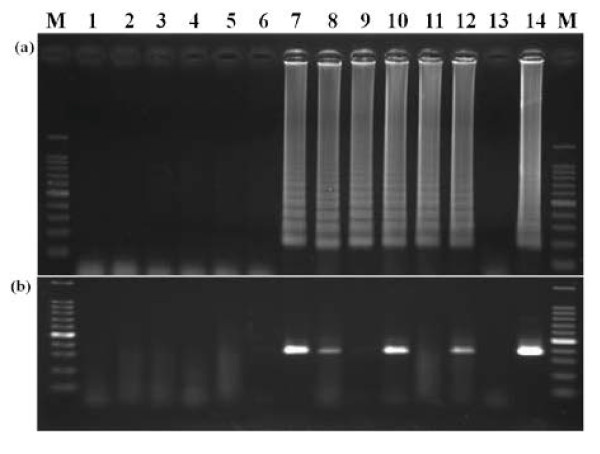
**Detection of field samples by RT-LAMP (a) and RT-PCR (b)**. M: 100-bp DNA ladder marker; 1-12: wheat samples collected from Yushan and Xiaqiao, Jiangsu Province: 11-6982, 11-6984, 11-6987, 11-7081, 11-7082, 11-7083, 11-7000, 11-7084, 11-7085,11-7086, 11-7087 and 11-7090, respectively; 13: healthy control; 14: positive control

Twenty-two samples from fields of different provinces were tested by RT-LAMP and RT-PCR. The cultivars including 11-6978,11-6982, 11-6984, 11-6987, 11-7081, 11-7082, 11-7083, 11-7000, 11-7084, 11-7085,11-7086, 11-7087 and 11-7090 and the positive wheat samples were collected in March 2011 from the virus-infected field of Jiangsu Province and kept at -20°C. The other samples were collected from wheat fields of Shandong Province, where there was no report of WYMV, in 2007. The detection results of RT-LAMP and RT-PCR were different. Eight of 22 samples were positive by RT-LAMP, whereas only five samples were positive by RT-PCR (half of agarose results were shown in Figure 7). This result confirmed the reliability and sensitivity of this RT-LAMP assay for routine detection.

## Discussion

WYMV could be detected by the RT-LAMP method using the designed four sets of primers (I-IV) and M-MLV reverse transcriptase instead of AMV reverse transcriptase under isothermal condition at 65°C for 80 min.

The primer set III seemed more sensitive than the others and was selected for further virus detection in this study. The RT-LAMP assay was performed at different times and virus was detected after 30-45 min by agarose gel analysis, considering the field application where the turbidity will be observed by naked eyes as the criteria to judge that the sample is negative or positive, the reaction condition at 65°C for 80 min was used according to previous studies and the results obtained here. The RT-LAMP method had detection sensitivity about 100 times more than RT-PCR in the present study, which was similar to that reported by Boubourakas [33]. Therefore, RT-LAMP was demonstrated to be a simple and time-saving method compared with RT-PCR for routine detection of WYMV, and it did not require specialized PCR and electrophoresis equipment. Due to the high level of detection sensitivity of the RT-LAMP method, careful and strict operation was necessary during the whole process to avoid false-positive results [34]. Other factors to consider are DNA smear and turbidity. Similar to products generated by PCR, DNA smear was present in the healthy control, which may due to an excess amount of RNA used in the reaction and some non-specific reactions [31]. If the DNA smear is strong enough, it can influence observation of turbidity. Turbidity can be observed directly, but DNA smear can also produce turbidity when the concentration of RNA template is sufficiently high. Therefore, when turbidity is used to differentiate positive and negative samples, the amount of RNA template should be maintained at a low level.

## Conclusion

In conclusion, this study developed the RT-LAMP assay for detecting WYMV. Compared with conventional RT-PCR, RT-LAMP yielded more accurate results, and was more convenient and less time-consuming especially for field detection. This method has potential application in early diagnosis and screening of resistant wheat varieties, to reduce the loss of yield.

## Competing interests

The authors declare that they have no competing interests.

## Authors' contributions

ZZ carried out most of the experiments and wrote the manuscript. XL performed the RT-PCR detection of CWMV and the DL, JY and CH conceived of the study and participated in its design and coordination. All authors read and approved the final manuscript.
